# Halogen, chalcogen, and hydrogen bonding in organoiodine cocrystals of heterocyclic thio­nes: imidazolidine-2-thione, 2-mercaptobenzimidazole, 2-mercapto-5-methyl­benzimidazole, 2-mercaptobenzoxazole, and 2-mercaptobenzo­thia­zole

**DOI:** 10.1107/S2053229622009548

**Published:** 2022-11-09

**Authors:** Spencer Watts, Andrew J. Peloquin, Madhushi Bandara, Colin D. McMillen, William T. Pennington

**Affiliations:** a Department of Chemistry, Clemson University, 219 Hunter Laboratories, Clemson, SC 29634, USA; University of Sheffield, United Kingdom

**Keywords:** crystal structure, halogen bonding, organoiodine, chalcogen bonding, hydro­gen bonding, thione

## Abstract

Through the combination of heterocyclic thio­nes with variation in the identity of the heterocyclic elements, namely, imidazolidine-2-thione, 2-mercaptobenzimid­azole, 2-mercapto-5-methyl­benz­imidazole, 2-mercaptobenzoxazole, and 2-mer­captobenzo­thia­zole with the common halogen-bond donors 1,2-, 1,3-, and 1,4-di­iodo­tetra­fluoro­benzene, 1,3,5-tri­fluoro­tri­iodo­benzene, and tetra­iodo­ethyl­ene, a series of 18 new crystalline structures were characterized. In most cases, N—H⋯S hydrogen bonding was observed, with these inter­actions in imidazole-con­taining structures typically resulting in two-dimensional motifs (*i.e.* ribbons). Lacking the second N—H group, the thia­zole and oxazole hydrogen bonding resulted in only dimeric pairs. C—I⋯S and C—I⋯I halogen bonding, as well as C=S⋯I chalcogen bonding, served to consolidate the packing by linking the hydrogen-bonding ribbons or dimeric pairs.

## Abstract 2

A series of 18 cocrystals were obtained through the combination of the heterocyclic mol­ecules imidazolidine-2-thione, 2-mercaptobenzimidazole, 2-mercapto-5-methyl­benz­imidazole, 2-mercaptobenzoxazole, and 2-mercaptobenzo­thia­zole with the common halogen-bond donors 1,2-, 1,3-, and 1,4-di­iodo­tetra­fluoro­benzene, 1,3,5-tri­fluoro­tri­iodo­benzene, and tetra­iodo­ethyl­ene. A rich series of hydrogen-, halogen-, and chalcogen-bonding inter­actions were observed.

## Introduction

Halogen and chalcogen bonding, defined by IUPAC as ‘a net attractive inter­action between an electrophilic region associated with…’ a halogen or chalcogen atom, respectively, ‘…in a mol­ecular entity and a nucleophilic region in another, or the same, mol­ecular entity (Desiraju *et al.*, 2013 ▸; Aakeroy *et al.*, 2019 ▸),’ has drawn increasing attention in recent years (Parisini *et al.*, 2011 ▸; Zhou *et al.*, 2010 ▸; Ajani *et al.*, 2015 ▸; Arman *et al.*, 2008 ▸; Aakeroy *et al.*, 2015 ▸; Metrangolo & Resnati, 2012 ▸; Cavallo *et al.*, 2016 ▸; Metrangolo *et al.*, 2005 ▸; Legon, 1998 ▸). Similar to hydrogen bonding, halogen bonding is strong, selective, and directional. Organic iodines are among the most commonly utilized halogen-bond donors (Corradi *et al.*, 2000 ▸), largely due to their greater polarizability. When paired with halogen-bond acceptor mol­ecules with a diversity of heteroatoms, the combined effects of halogen, chalcogen, and hydrogen bonding can be revealed. Imidazoles, thia­zoles, and oxazoles are ideal systems to study in this regard.

**Figure 1:**
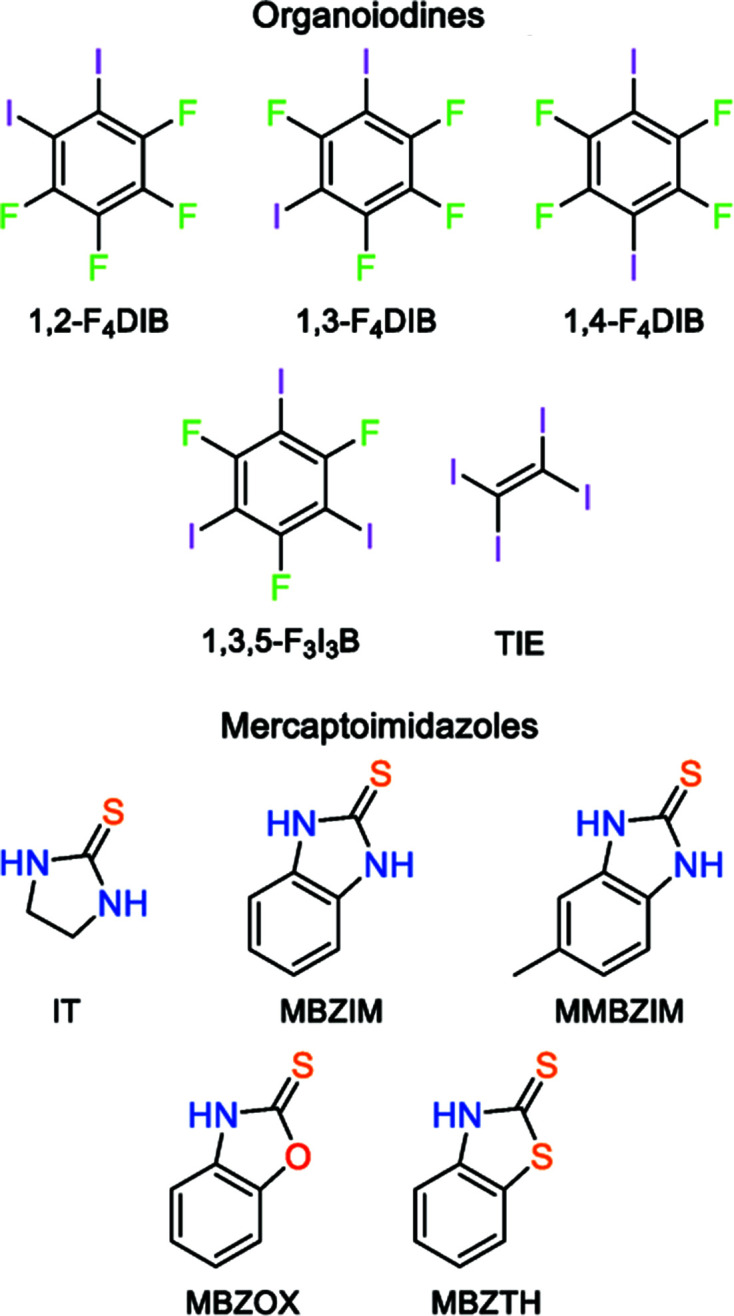
Organoiodines and mercapto­imidazoles utilized in this study.

Benzimidazole, and its derivatives, have been investigated for a diverse range of biological applications, including in the treatment of tuberculosis (Foks *et al.*, 2006 ▸), as anti­microbial agents (Alasmary *et al.*, 2015 ▸), and also as analgesic and anti-inflammatory com­pounds (Achar *et al.*, 2010 ▸; Fletcher *et al.*, 2006 ▸). These mercaptobenzimidazoles, thia­zoles, and oxazoles have also seen significant utilization as ligands in transition-metal com­plexes. Providing some insight into the role of heteroatoms in differing positions, of the 31 crystal structures containing 2-mercaptobenzo­thia­zole (**MBZTH**) and a transition metal currently deposited with the Cambridge Structural Database (CSD; Groom *et al.*, 2016 ▸), all demonstrate metal coordination through the thione S atom and not the thia­zole S atom. They range from simple species, such as (2-mer­cap­to­benzo­thia­zole)bis­(tri­phenyl­phosphine)silver(I) iodide (Banti *et al.*, 2014 ▸), to more com­plex copper and ruthenium com­plexes (Zhou *et al.*, 2013*a* ▸; Zafar *et al.*, 2019 ▸). Similarly, the mercaptobenzimidazole (or benzimidazole­thione) derivatives present an inter­esting field of study for their potential inter­molecular inter­actions in halogen-bonding systems (Fig. 1 ▸). In these systems, hydrogen, halogen, and chalcogen bonding are all viable inter­molecular inter­actions, and structural studies of the cocrystals can be useful in determining which inter­actions are preferred as the organo­iodine and the heterocyclic systems are varied.

Our group has recently been inter­ested in the role of the S atom in I⋯S halogen- and chalcogen-bonding inter­actions as a crystal design tool, as well as their roles in the formation of deep eutectic solvents derived from halogen bonding (Peloquin *et al.*, 2021*a* ▸,*b* ▸,*c* ▸,*d* ▸, 2022 ▸). Herein, we report the solid-state structures of 18 new cocrystals derived from the combination of the heterocyclic mol­ecules imidazolidine-2-thione (**IT**), 2-mercaptobenzimidazole (**MBZIM**), 2-mercapto-5-methyl­benz­imidazole (**MMBZIM**), 2-mercaptobenzoxazole (**MBZOX**), and 2-mercaptobenzo­thia­zole (**MBZTH**) with the organic halo­gen-bond donors 1,2-di­iodo­tetra­fluoro­benzene (**1,2-F_4_DIB**), 1,3-di­iodo­tetra­fluoro­benzene (**1,3-F_4_DIB**), 1,4-tetra­fluoro­benzene (**1,4-F_4_DIB**), 1,3,5-tri­fluoro-2,4,6-tri­iodo­ben­zene (**1,3,5-F_3_I_3_B**), and tetra­iodo­ethyl­ene (**TIE**). This diverse pool of substrates yielded structures with the crystal packing dominated by N—H⋯S hydrogen bonding, leading to thio­amide dimers, with longer-range packing motifs created through C—I⋯S and C—I⋯I halogen bonding, as well as the occasional C=S⋯I chalcogen bond.

## Experimental

### Materials and instrumentation

For single-crystal X-ray analysis, crystals were mounted on low background cryogenic loops using paratone oil. Data were collected using Mo *K*α radiation (λ = 0.71073 Å) on a Bruker D8 Venture diffractometer with an Incoatec Iµs microfocus source and a Photon 2 detector.

### Preparation of cocrystals

Cocrystals were synthesized using imidazolidine-2-thione (TCI Americas, 98%), 2-mercaptobenzimidazole (Acros, 98%), 2-mercapto-5-methyl­benzimidazole (Acros, 99%), 2-mer­captobenzoxazole (Acros, 99%), 2-mercaptobenzo­thia­zole (Acros, 98%), 1,2-di­iodo­tetra­fluoro­benzene (Synquest Lab­oratories, 99%), 1,3-di­iodo­tetra­fluoro­benzene (Synquest Lab­oratories, 97%), 1,4-tetra­fluoro­benzene (Synquest Lab­ora­tories, 97%), 1,3,5-tri­fluoro-2,4,6-tri­iodo­benzene (Synquest Laboratories, 99%), and tetra­iodo­ethyl­ene (Santa Cruz Biotechnologies, 98%). Solvents were obtained from Fisher Scientific. All materials were used as received without further purification. Crystals were formed by slow evaporation under ambient conditions (20–23 °C). Methanol was utilized for the majority of cocrystal preparations; however, if this was not successful, acetone or ethyl acetate was utilized.

#### 2(**IT**)·(1,3-F_4_DIB)

Imidazolidine-2-thione (50 mg, 0.489 mmol) and 1,3-di­iodo­tetra­fluoro­benzene (196 mg, 0.489 mmol) were weighed into a 20 ml glass vial. Methanol (10 ml) was added and the mixture was stirred until a clear solution was obtained. The solvent was allowed to evaporate slowly and colorless needle-like crystals of 2(**IT**)·(**1,3-F_4_DIB**) were obtained after 3 d.

#### (**IT**)·(**1,3,5-F_3_I_3_B**)

Imidazolidine-2-thione (50 mg, 0.489 mmol) and 1,3,5-tri­fluoro-2,4,6-tri­iodo­benzene (249 mg, 0.489 mmol) were weighed into a 20 ml glass vial. Methanol (10 ml) was added and the mixture was stirred until a clear solution was obtained. The solvent was allowed to evaporate slowly and colorless needle-like crystals of (**IT**)·(**1,3,5-F_3_I_3_B**) were obtained after 4 d.

#### 4(**MBZIM**)·3(1,3-F_4_DIB)

2-Mercaptobenzimidazole (34 mg, 0.227 mmol) and 1,3-di­iodo­tetra­fluoro­benzene (49 mg, 0.122 mmol) were weighed into a 20 ml glass vial. Methanol (10 ml) was added and the mixture was stirred until a clear solution was obtained. The solvent was allowed to evaporate slowly and colorless needle-like crystals of 4(**MBZIM**)·3(**1,3-F_4_DIB**) were obtained after 4 d.

#### (**MBZIM**)·(1,4-F_4_DIB)

2-Mercaptobenzimidazole (19 mg, 0.126 mmol) and 1,4-di­iodo­tetra­fluoro­benzene (50 mg, 0.124 mmol) were weighed into a 20 ml glass vial. Methanol (10 ml) was added and the mixture was stirred until a clear solution was obtained. The solvent was allowed to evaporate slowly and colorless plate-like crystals of (**MBZIM**)·(**1,4-F_4_DIB**) were obtained after 3 d.

#### (**MBZIM**)·(**TIE**)

2-Mercaptobenzimidazole (30 mg, 0.200 mmol) and tetra­iodo­ethyl­ene (55 mg, 0.103 mmol) were weighed into a 20 ml glass vial. Ethyl acetate (15 ml) was added and the mixture was stirred until a clear solution was obtained. The solvent was allowed to evaporate slowly and colorless tabular crystals of (**MBZIM**)·(**TIE**) were obtained after 7 d.

#### (**MMBZIM**)·(1,2-F_4_DIB)

2-Mercapto-5-methyl­benz­imidazole (20 mg, 0.122 mmol) and 1,2-di­iodo­tetra­fluoro­benzene (48 mg, 0.119 mmol) were weighed into a 20 ml glass vial. Methanol (10 ml) was added and the mixture was stirred until a clear solution was obtained. The solvent was allowed to evaporate slowly and colorless columnar crystals of (**MMBZIM**)·(**1,2-F_4_DIB**) were obtained after 3 d.

#### 2(**MMBZIM**)·(1,4-F_4_DIB)·2(H_2_O)

2-Mercapto-5-methyl­benzimidazole (40 mg, 0.244 mmol) and 1,4-di­iodo­tetra­fluoro­benzene (51 mg, 0.127 mmol) were weighed into a 20 ml glass vial. Methanol (10 ml) was added and the mixture was stirred until a clear solution was obtained. The solvent was allowed to evaporate slowly and colorless plate-like crystals of 2(**MMBZIM**)·(**1,4-F_4_DIB**)·2(**H_2_O**) were obtained after 3 d.

#### (**MMBZIM**)·(**1,3,5-F_3_I_3_B**)

2-Mercapto-5-methyl­benz­imidazole (31 mg, 0.189 mmol) and 1,3,5-tri­fluoro-2,4,6-tri­iodo­benzene (50 mg, 0.098 mmol) were weighed into a 20 ml glass vial. Methanol (10 ml) was added and the mixture was stirred until a clear solution was obtained. The solvent was allowed to evaporate slowly and colorless needle-like crystals of (**MMBZIM**)·(**1,3,5-F_3_I_3_B**) were obtained after 4 d.

#### (**MBZOX**)·(1,2-F_4_DIB)

2-Mercaptobenzoxazole (20 mg, 0.132 mmol) and 1,2-di­iodo­tetra­fluoro­benzene (102 mg, 0.254 mmol) were weighed into a 20 ml glass vial. Methanol (10 ml) was added and the mixture was stirred until a clear solution was obtained. The solvent was allowed to evaporate slowly and colorless needle-like crystals of (**MBZOX**)·(**1,2-F_4_DIB**) were obtained after 3 d.

#### (**MBZOX**)·(1,3-F_4_DIB)

2-Mercaptobenzoxazole (19 mg, 0.126 mmol) and 1,3-di­iodo­tetra­fluoro­benzene (104 mg, 0.259 mmol) were weighed into a 20 ml glass vial. Acetone (10 ml) was added and the mixture was stirred until a clear solution was obtained. The solvent was allowed to evaporate slowly and colorless columnar crystals of (**MBZOX**)·(**1,3-F_4_DIB**) were obtained after 2 d.

#### 2(**MBZOX**)·(1,4-F_4_DIB)

2-Mercaptobenzoxazole (40 mg, 0.265 mmol) and 1,4-di­iodo­tetra­fluoro­benzene (50 mg, 0.124 mmol) were weighed into a 20 ml glass vial. Acetone (10 ml) was added and the mixture was stirred until a clear solution was obtained. The solvent was allowed to evaporate slowly and colorless columnar crystals of 2(**MBZOX**)·(**1,4-F_4_DIB**) were obtained after 2 d.

#### (**MBZOX**)·(**1,3,5-F_3_I_3_B**)

2-Mercaptobenzoxazole (15 mg, 0.099 mmol) and 1,3,5-tri­fluoro-2,4,6-tri­iodo­benzene (50 mg, 0.098 mmol) were weighed into a 20 ml glass vial. Acetone (10 ml) was added and the mixture was stirred until a clear solution was obtained. The solvent was allowed to evaporate slowly and colorless columnar crystals of (**MBZOX**)·(**1,3,5-F_3_I_3_B**) were obtained after 1 d.

#### 3(**MBZTH**)·4(1,2-F_4_DIB)

2-mercaptobenzo­thia­zole (21 mg, 0.126 mmol) and 1,2-di­iodo­tetra­fluoro­benzene (103 mg, 0.256 mmol) were weighed into a 20 ml glass vial. Methanol (10 ml) was added and the mixture was stirred until a clear solution was obtained. The solvent was allowed to evaporate slowly and colorless plate-like crystals of 3(**MBZTH**)·4(**1,2-F_4_DIB**) were obtained after 3 d.

#### (**MBZTH**)·(1,3-F_4_DIB)

2-Mercaptobenzo­thia­zole (24 mg, 0.143 mmol) and 1,3-di­iodo­tetra­fluoro­benzene (50 mg, 0.124 mmol) were weighed into a 20 ml glass vial. Methanol (10 ml) was added and the mixture was stirred until a clear solution was obtained. The solvent was allowed to evaporate slowly and colorless plate-like crystals of (**MBZTH**)·(**1,3-F_4_DIB**) were obtained after 3 d.

#### (**MBZTH**)·2(1,3-F_4_DIB)

2-Mercaptobenzo­thia­zole (22 mg, 0.132 mmol) and 1,3-di­iodo­tetra­fluoro­benzene (98 mg, 0.244 mmol) were weighed into a 20 ml glass vial. Methanol (10 ml) was added and the mixture was stirred until a clear solution was obtained. The solvent was allowed to evaporate slowly and colorless tabular crystals of (**MBZTH**)·2(**1,3-F_4_DIB**) were obtained after 4 d.

#### 2(**MBZTH**)·(1,4-F_4_DIB)

2-Mercaptobenzo­thia­zole (46 mg, 0.275 mmol) and 1,4-di­iodo­tetra­fluoro­benzene (50 mg, 0.124 mmol) were weighed into a 20 ml glass vial. Acetone (10 ml) was added and the mixture was stirred until a clear solution was obtained. The solvent was allowed to evaporate slowly and colorless needle-like crystals of 2(**MBZTH**)·(**1,4-F_4_DIB**) were obtained after 2 d.

#### (**MBZTH**)·(**1,3,5-F_3_I_3_B**)

2-Mercaptobenzo­thia­zole (32 mg, 0.191 mmol) and 1,3,5-tri­fluoro-2,4,6-tri­iodo­benzene (50 mg, 0.098 mmol) were weighed into a 20 ml glass vial. Acetone (10 ml) was added and the mixture was stirred until a clear solution was obtained. The solvent was allowed to evaporate slowly and colorless tabular crystals of (**MBZTH**)·(**1,3,5-F_3_I_3_B**) were obtained after 2 d.

#### (**MBZTH**)·(**TIE**)

2-Mercaptobenzo­thia­zole (33 mg, 0.197 mmol) and tetra­iodo­ethyl­ene (50 mg, 0.094 mmol) were weighed into a 20 ml glass vial. Methanol (15 ml) was added and the mixture was stirred with gentle heating until a clear solution was obtained. The solvent was allowed to evaporate slowly and colorless block-like crystals of (**MBZTH**)·(**TIE**) were obtained after 5 d.

### Refinement

Crystal data, data collection and structure refinement details are summarized in Table 1 ▸. H atoms on C atoms were calculated in idealized positions riding on their parent atoms, with C—H = 0.98 Å and *U*_iso_(H) = 1.5*U*_eq_(C) for methyl H atoms, and C—H = 0.95 Å and *U*_iso_(H) = 1.2*U*_eq_(C) for other H atoms. H atoms on heteroatoms were located in difference Fourier maps and refined isotropically, utilizing appropriate restraints [N—H = 0.86 (2) Å] where necessary to maintain chemically reasonable geometries. The H atoms of the water molecule in 2(**MMBZIM**)·(**1,4-F_4_DIB**)·2(**H_2_O**) were modeled in a disordered arrangement due to symmetry considerations.

## Results and discussion

### Cocrystals of imidazolidine-2-thione (**IT**)

**Figure 2:**
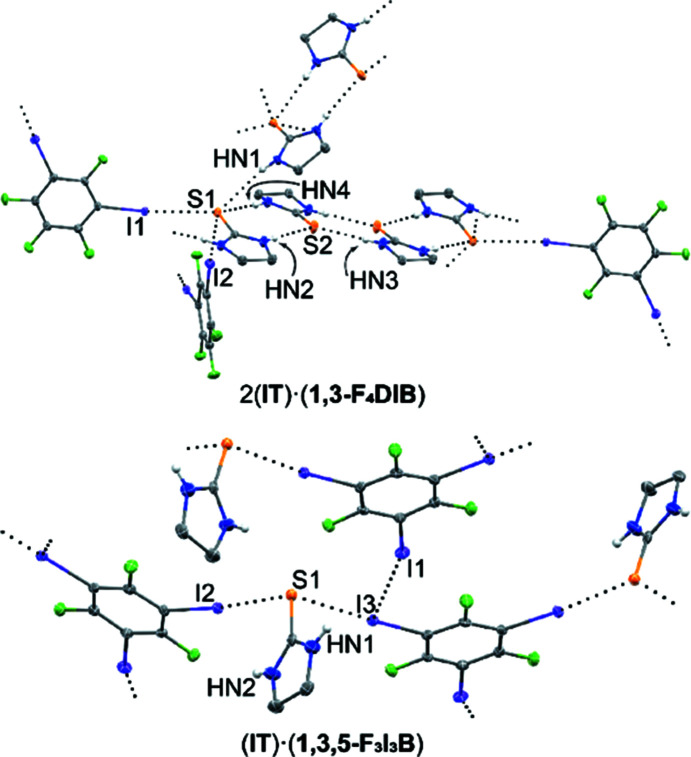
Cocrystalline structures containing **IT**. Hydrogen and halogen bonding are indicated by black dotted lines. Displacement ellipsoids are drawn at the 50% probability level. H atoms, except those bound to N atoms, have been omitted for clarity.

The smallest of the sulfur-containing com­pounds within this study, imidazolidine-2-thione, contains a thio­urea functionality within a five-membered saturated ring. The first cocrystal formed with this com­pound in the present study is 2(**IT**)·(**1,3-F_4_DIB**), which was refined in the ortho­rhom­bic space group *Pbcn* with two unique mol­ecules of **IT** and one mol­ecule of **1,3-F_4_DIB** in the asymmetric unit (Fig. 2 ▸). As is common in thio­urea-containing structures, a pair of N—H⋯S hydrogen bonds links thio­urea mol­ecules, in this case, into tetra­meric units (Table 2 ▸) (Peloquin *et al.*, 2021*d* ▸, 2022 ▸). This is in contrast to the formation of hydrogen-bonded ribbons and discrete dimers, which are formed in the previously published 2(**IT**)·(**1,2-F_4_DIB**) and (**IT**)·2(**1,2-F_4_DIB**) cocrystals, respectively (Happonen *et al.*, 2021 ▸). Tetra­meric units align into staggered stacks in the *b* direction. These stacks are separated by additional tetra­meric units, with the planes of the tetra­mers inclined by approximately 64°. This arrangement of inclined hydrogen-bonding units is also observed in the dimeric units of (**IT**)·(**1,4-F_4_DIB**) (Happonen *et al.*, 2021 ▸). At the end of each tetra­mer, the remaining N—H hydrogen serves to link to the next inclined tetra­mer *via* N—H⋯S hydrogen bonding. The S atom at this end, S1, acts as a C—I⋯S halogen-bond acceptor to two different **1,3-F_4_DIB** mol­ecules (Table 3 ▸). These halogen-bonding inter­actions link adjacent stacks of tetra­mers in the *c* direction. The second **IT**-containing co­crystal of this study, (**IT**)·(**1,3,5-F_3_I_3_B**), was refined in the ortho­rhom­bic space group *Pbca* with one mol­ecule each of **IT** and **1,3,5-F_3_I_3_B** in the asymmetric unit. This structure represents the only example within this study without N—H⋯S hydrogen bonding (Table 4 ▸). Instead, C—I⋯S halogen bonding occurs between alternating mol­ecules of **IT** and **1,3,5-F_3_I_3_B** to form chains propagating in the *c* direction. The third I atom of **1,3,5-F_3_I_3_B**, which does not participate in significant inter­actions with sulfur, instead serves to link chains in the *ac* plane *via* C—I⋯I halogen bonding.

### Cocrystals of 2-mercaptobenzimidazole (**MBZIM**)

**Figure 3:**
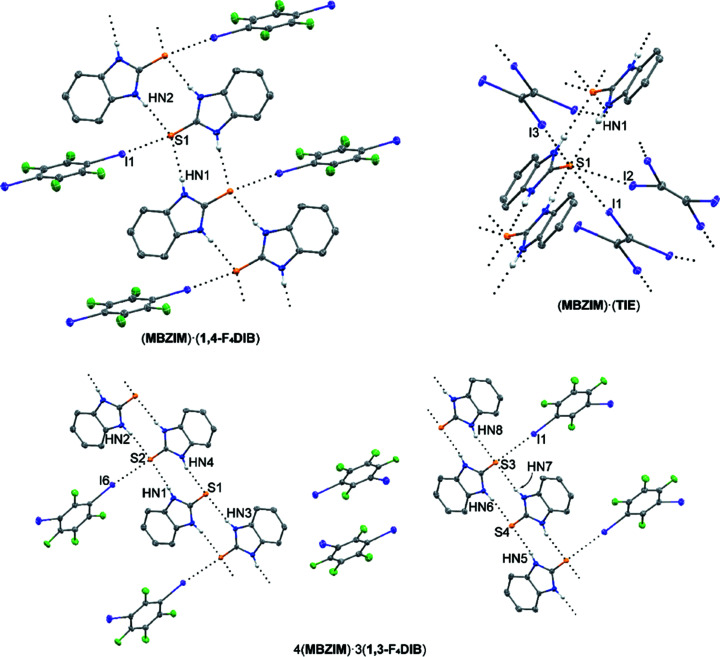
Cocrystal structures containing **MBZIM**. Hydrogen and halogen bonding are indicated by black dotted lines. Displacement ellipsoids are drawn at the 50% probability level. H atoms, except those bound to N atoms, have been omitted for clarity.

Moving to the larger thio­urea-containing mol­ecule 2-mer­cap­tobenzimidazole (**MBZIM**) yielded three new structures dominated by co-operative hydrogen and halogen bonding (Fig. 3 ▸). With **1,3-F_4_DIB**, the cocrystalline structure of 4(**MBZIM**)·3(**1,3-F_4_DIB**) was obtained in the triclinic space group *P* 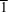 , with four unique mol­ecules of **MBZIM** and three mol­ecules of **1,3-F_4_DIB** in the asymmetric unit. In this structure, hydrogen bonding between thio­urea mol­ecules contributes to the formation of ribbons propagating along the *a* axis (Table 5 ▸). Two of the three **1,3-F_4_DIB** mol­ecules are pendants along these chains, linked *via* C—I⋯S. The second I atom of these particular **1,3-F_4_DIB** mol­ecules does not contribute to significant halogen- or chalcogen-bonding inter­actions. This hydrogen-bonding thio­urea ribbon with halogen-bonding pendants is analogous to that observed in (**MBZIM**)·(**1,2-F_4_DIB**) (Arman *et al.*, 2008 ▸, 2010 ▸). The final unique **1,3-F_4_DIB** mol­ecule lies between the ring planes of the pendant mol­ecules of **1,3-F_4_DIB**, contributing to only weak C—I⋯H, C—F⋯H, and C—F⋯F—C inter­actions. The combination of **MBZIM** and **1,4-F_4_DIB** resulted in the (**MBZIM**)·(**1,4-F_4_DIB**) cocrystal, refined in the monoclinic space group *P*2_1_/*c*, with one mol­ecule each of both **MBZIM** and **1,4-F_4_DIB** in the asymmetric unit. Just as in 4(**MBZIM**)·3(**1,3-F_4_DIB**), the structure of (**MBZIM**)·(**1,4-F_4_DIB**) consists of ribbons of **MBZIM** mol­ecules propagating in the *c* direction, formed through thio­urea hydrogen bonding (Table 6 ▸). Mol­ecules of **1,4-F_4_DIB** act as pendants along these ribbons, linked *via* C—I⋯S halogen bonding.

With four I atoms available, tetra­iodo­ethyl­ene (**TIE**) often enables structural motifs that are different from the typical aromatic halogen-bond donors. The cocrystal (**MBZIM**)·(**TIE**) was refined in the ortho­rhom­bic space group *Pnma*, with one mol­ecule each of **MBZIM** and **TIE** in the asymmetric unit. As in the previous examples, mol­ecules of **MBZIM** form infinite ribbons through thio­urea hydrogen bonding (Table 7 ▸). Three of the four I atoms of **TIE** function as C—I⋯S halogen-bond donor atoms to link these ribbons, creating a three-dimensional framework through the combination of hydrogen and halogen bonding. The fourth I atom participates in a C—I⋯π inter­action [I⋯π = 3.351 (3) Å] to reinforce the frame­work.

### Cocrystals of 2-mercapto-5-methyl­benzimidazole (**MMBZIM**)

**Figure 4:**
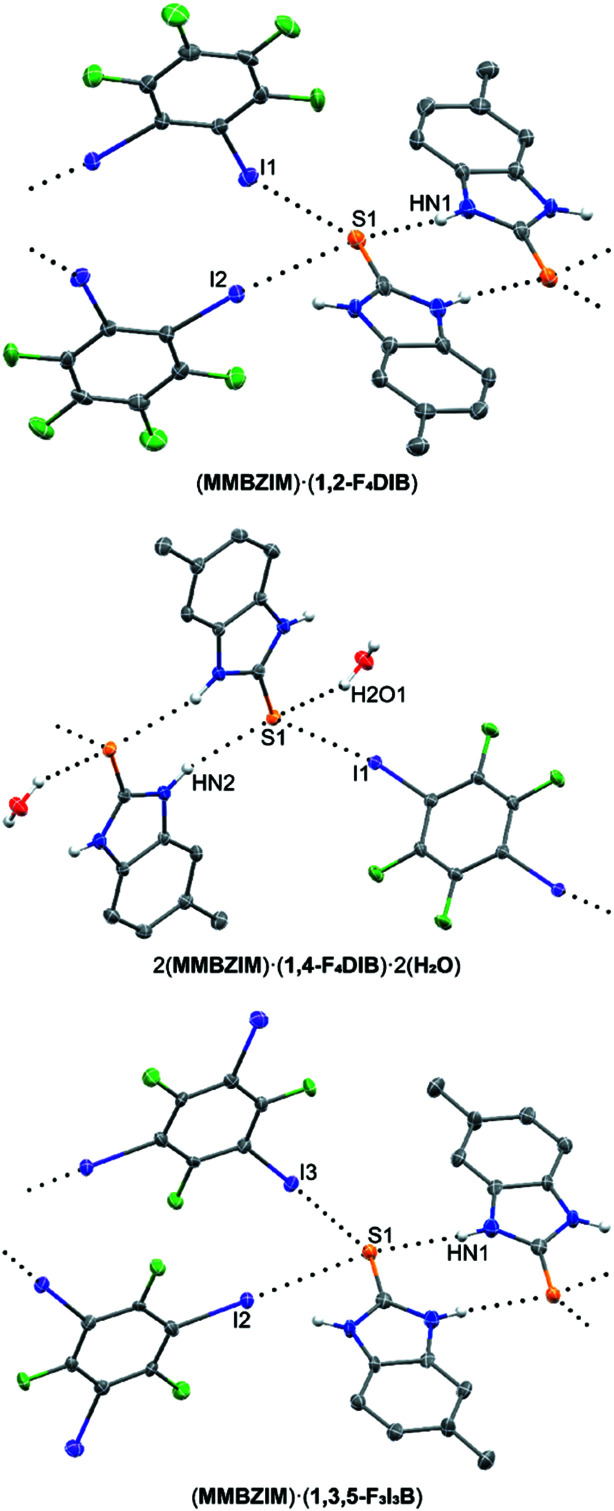
Cocrystalline structures containing **MMBZIM**. Hydrogen and halogen bonding are indicated by black dotted lines. Displacement ellipsoids are drawn at the 50% probability level. H atoms, except those bound to N atoms, have been omitted for clarity.

Adding a methyl group to **MBZIM**, resulting in 2-mercapto-5-methyl­benzimidazole (**MMBZIM**), induces significant changes to the overall hydrogen- and halogen-bonding motifs. The structural literature of this substrate is limited, having only been characterized by single-crystal X-ray diffraction when acting as a ligand for transition metals coordinating through its S atom (Lin *et al.*, 2017 ▸; Ozturk *et al.*, 2009 ▸; Mitra *et al.*, 2012 ▸). The first halogen-bonded cocrystal of **MMBZIM** in this study, (**MMBZIM**)·(**1,2-F_4_DIB**), was refined in the triclinic space group *P* 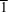 , with one mol­ecule each of **MMBZIM** and **1,2-F_4_DIB** in the asymmetric unit (Fig. 4 ▸). A discrete hydrogen-bonded dimer of two **MMBZIM** mol­ecules is observed, in contrast to the infinite ribbons in (**MBZIM**)·(**1,2-F_4_DIB**) and most of the cocrystals in the present study (Table 8 ▸). Two mol­ecules of **1,2-F_4_DIB** per **MMBZIM** molecule link the dimers *via* C—I⋯S halogen bonds, leading to the formation of chains along the *c* axis.

Isolated as a hydrated cocrystal from adventitious water, 2(**MMBZIM**)·(**1,4-F4DIB**)·2(**H_2_O**) crystallizes in the triclinic space group *P* 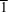 with one mol­ecule each of **MMBZIM** and **H_2_O**, as well as half a mol­ecule of **1,4-F_4_DIB**, in the asymmetric unit. All attempts to obtain an nonhydrated cocrystal with **1,4-F_4_DIB** were unsuccessful, suggesting the packing arrangement formed strictly by halogen bonding contains small but meaningful voids that must be occupied by the water mol­ecule. Discrete hydrogen-bonded dimers are again observed by hydrogen bonding of the thio­amides (Table 9 ▸). Differing from (**MMBZIM**)·(**1,2-F_4_DIB**), with two halogen bonds to each S atom, 2(**MMBZIM**)·(**1,4-F4DIB**)·2(**H_2_O**) utilizes one C—I⋯S halogen bond and one O—H⋯S hydro­gen bond at each S atom. It is the halogen bonding that contributes to the formation of infinite chains by linking the discrete dimers. The water mol­ecule also acts as an N—H⋯O hydrogen-bond acceptor from the N atom that does not participate in thio­amide hydrogen bonding and so is an inter­mediate linker facilitating the formation of an expanded thio­amide ribbon motif.

Finally, the combination of **1,3,5-F_3_I_3_B** and **MMBZIM** resulted in the cocrystal (**MMBZIM**)·(**1,3,5-F_3_I_3_B**), refined in the monoclinic space group *P*2_1_/*c* with one unique mol­ecule of each com­ponent in the asymmetric unit. The overall packing motif in this structure is strikingly similar to that in (**MMBZIM**)·(**1,2-F_4_DIB**). Two mol­ecules of **MMBZIM** form dimeric pairs through hydrogen bonding of the thio­amides (Table 10 ▸). The remaining N—H hydrogens are involved in weak N—H⋯I hydrogen bonds [H⋯I = 3.02 (8) Å]. A pair of C—I⋯S halogen bonds occurs at each S atom, contributing to chains propagating in the *a* direction. The third I atom is oriented as a potential acceptor for a C—F⋯I inter­action, though the inter­action distance is very near the sum of the van der Waals radii and it is unclear if there is a significant attraction to this inter­action. Given the similar motifs of (**MMBZIM**)·(**1,3,5-F_3_I_3_B**) to (**MMBZIM**)·(**1,2-F_4_DIB**), it may be that the C—F⋯I contact is merely coincident within the motif formed by the N—H⋯S and C—I⋯S inter­actions.

### Cocrystals of 2-mercaptobenzoxazole (**MBZOX**)

**Figure 5:**
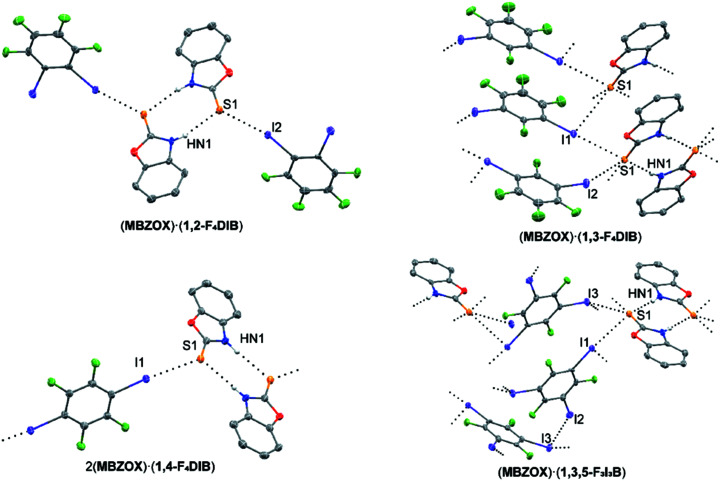
Cocrystalline structures containing **MBZOX**. Hydrogen and halogen bonding are indicated by black dotted lines. Displacement ellipsoids are drawn at the 50% probability level. H atoms, except those bound to N atoms, have been omitted for clarity.

While infinite ribbons commonly formed through hydrogen bonding of the thio­ureas in **MBZIM**, substituting one secondary N atom for an O atom in 2-mercaptobenzoxazole (**MBZOX**) allows for the study of the structural motifs when only dimers can form through hydrogen bonding (Fig. 5 ▸). The structural literature surrounding **MBZOX** is sparse, limited to three reports of it acting as a ligand through the S atom in transition-metal com­plexes (McFarlane *et al.*, 1998 ▸; Nakahodo *et al.*, 2000 ▸; Mitra *et al.*, 2012 ▸) and its reaction with diiodine (Cristiani *et al.*, 1995 ▸). Combined with **1,2-F_4_DIB**, the cocrystalline structure of (**MBZOX**)·(**1,2-F_4_DIB**) was refined in the monoclinic space group *P*2_1_/*n*, with one unique mol­ecule each of **MBZOX** and **1,2-F_4_DIB** in the asymmetric unit. Here, a hydrogen-bonding thio­amide dimer is formed (Table 11 ▸), with each S atom acting as an acceptor to a single C—I⋯S halogen bond. The second I atom does not contribute to an additional halogen bond, instead being involved in a weak C—I⋯π inter­action. This discrete four-mol­ecule unit formed through hydrogen and halogen bonding stands in stark contrast to the infinite hydrogen-bonding ribbon with pendant halogen-bonded **1,2-F_4_DIB** mol­ecules observed in (**MBZIM**)·(**1,2-F_4_DIB**). The pattern of inter­actions in (**MBZOX**)·(**1,3-F_4_DIB**), which crystallizes in the monoclinic space group *P*2_1_/*c*, with one mol­ecule each of **MBZOX** and **1,3-F_4_DIB** in the asymmetric unit, is more com­plex. Thio­amide hydrogen-bonding dimers are once again observed (Table 12 ▸). These dimers stack along the *b* axis. Mol­ecules of **1,3-F_4_DIB** link neighboring stacks of dimers in the *a* direction. One of the I atoms serves as both a C—I⋯S halogen-bond donor and a C=S⋯I chalcogen-bond acceptor. The combination of halo­gen, chalcogen, and hydrogen-bonding results in the formation of a two-dimensional motif of inter­molecular inter­actions. In 2(**MBZOX**)·(**1,4-F_4_DIB**), which was refined in the monoclinic space group *C*2/*c*, with one mol­ecule of **MBZOX** and one-half of a mol­ecule of **1,4-F_4_DIB**, the packing motif is more reminiscent of its **MMBZIM** analogue. Thio­amide hydrogen-bonding dimers are linked into chains through C—I⋯S halogen bonding (Table 13 ▸). The final example in the **MBZOX** series, (**MBZOX**)·(**1,3,5-F_3_I_3_B**), was refined in the monoclinic space group *P*2_1_/*c*, with one mol­ecule each of **MBZOX** and **1,3,5-F_3_I_3_B** in the asymmetric unit. Much of the packing is similar to (**MBZOX**)·(**1,3-F_4_DIB**), with thio­amide hydrogen-bonding dimers stacking in the *b* direction (Table 14 ▸). Neighboring stacks are linked along the *a* axis by both C—I⋯S halogen bonding and a C=S⋯I chalcogen bond to again form a two-dimensional substructure. In this instance though, the third I atom of **1,3,5-F_3_I_3_B** acts as a C—I⋯I halogen-bond donor, further consolidating the packing in the *c* direction to form a three-dimensional framework. In all cases of these **MBZOX** cocrystals, hydrogen- and halogen-bonding preference is given toward the thione S atom as the acceptor rather than the O atom of the heterocycle.

### Cocrystals of 2-mercaptobenzo­thia­zole (**MBZTH**)

**Figure 6:**
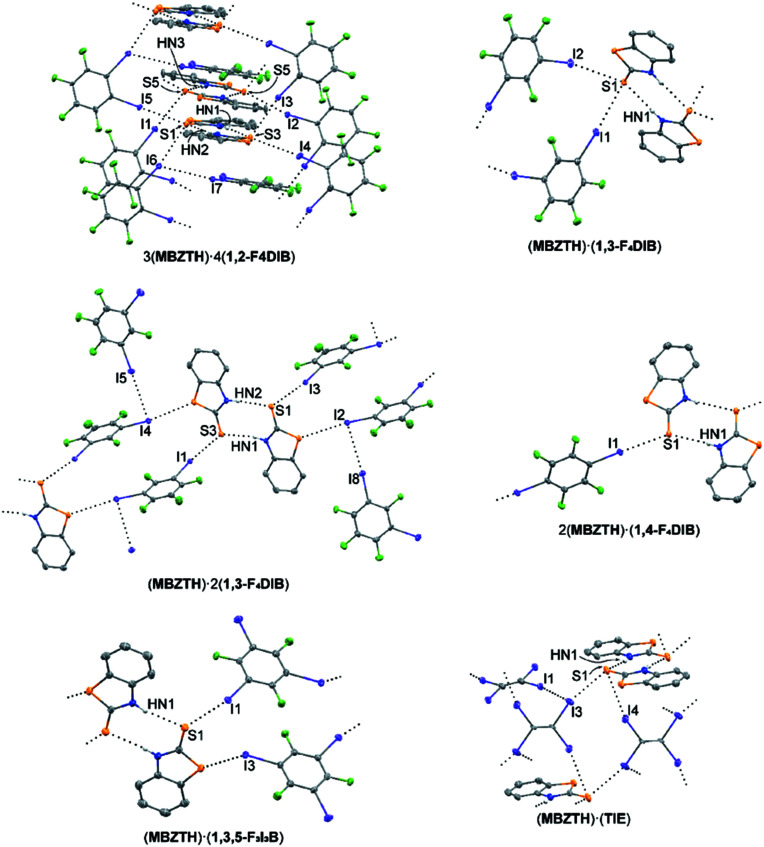
Cocrystalline structures containing **MBZTH**. Hydrogen and halogen bonding are indicated by black dotted lines. Displacement ellipsoids are drawn at the 50% probability level. H atoms, except those bound to N atoms, have been omitted for clarity.

As with **MBZOX**, 2-mercaptobenzo­thia­zole lacks the thio­urea functionality to allow for the formation of infinite ribbons through hydrogen bonding; however, the additional S atom can potentially act in either halogen- or chalcogen-bonding inter­actions (Fig. 6 ▸). Just as with **MBZOX**, the prior structural literature is dominated by examples of **MBZTH** acting as a ligand in transition-metal com­plexes (Aslanidis *et al.*, 2002 ▸; Zhou *et al.*, 2013*b* ▸; Hadjikakou & Kubicki, 2000 ▸) or reactions with dihalides (Daga *et al.*, 2002 ▸; Koskinen *et al.*, 2015*a* ▸,*b* ▸). The first and most com­plex of the **MBZTH** structures obtained, 3(**MBZTH**)·4(**1,2-F_4_DIB**), crystallized in the triclinic space group *P* 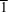 , with three mol­ecules of **MBZTH** and four mol­ecules of **1,2-F_4_DIB** in the asymmetric unit. Thio­amide dimers stack along the *a* axis (Table 15 ▸), with one mol­ecule of **1,2-F_4_DIB** within alternating layers. The remaining mol­ecules of **1,2-F_4_DIB** are oriented approximately perpendicular to the thio­amide dimers, linking layers of the stack through a series of C—I⋯S halogen bonds. The intra-stack mol­ecule of **1,2-F_4_DIB** is also linked to a mol­ecule of **1,2-F_4_DIB** on the edge of the stack through a C—I⋯I halogen bond. This com­plex series of inter­actions ultimately forms a three-dimensional framework.

The packing motif of (**MBZTH**)·(**1,3-F_4_DIB**), refined in the triclinic space group *P* 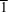 , with one mol­ecule each of **MBZTH** and **1,3-F_4_DIB** within the asymmetric unit, is similar to that of (**MMBZIM**)·(**1,2-F_4_DIB**) and (**MMBZIM**)·(**1,3,5-F_3_I_3_B**). Thio­­amide hydrogen-bonding dimers (Table 16 ▸) are linked by a pair of unique C—I⋯S halogen bonds to the thione S atom, forming chains in the *c* direction. Crystallizing in the monoclinic space group *P*2_1_, the asymmetric unit of (**MBZTH**)·2(**1,3-F_4_DIB**) contains two unique mol­ecules of **MBZTH** and four mol­ecules of **1,3-F_4_DIB**. In this case, the thio­amide hydrogen-bonding dimers (Table 17 ▸) are linked by mol­ecules of **1,3-F_4_DIB** *via* C—I⋯S halogen bonding to form chains. These inter­actions occur to the thione and thia­zole S atoms, with the inter­action to the thione S atom occurring at a distance approximately 0.35 Å shorter than to the thia­zole S atom. The remaining two mol­ecules of **1,3-F_4_DIB** are located as pendants along the chain, linked by C—I⋯I halogen bonding.

The packing motif of 2(**MBZTH**)·(**1,4-F_4_DIB**), refined in the monoclinic space group *P*2_1_/*n*, with one com­plete mol­ecule of **MBZTH** and one-half of a mol­ecule of **1,4-F_4_DIB** in the asymmetric unit, is similar to that of 2(**MBZOX**)·(**1,4-F_4_DIB**). Thio­amide hydrogen-bonding dimers (Table 18 ▸) are linked into chains *via* C—I⋯S halogen bonding to the thione S atom. As the final example with an aromatic halogen-bond donor, (**MBZTH**)·(**1,3,5-F_3_I_3_B**) was obtained in the monoclinic space group *P*2_1_/*c*, with one unique mol­ecule each of both **MBZTH** and **1,3,5-F_3_I_3_B** in the asymmetric unit. The primary packing motif is similar to that of (**MBZTH**)·2(**1,3-F_4_DIB**), with the thio­amide hydrogen-bonding dimers (Table 19 ▸) linked into chains by C—I⋯S halogen bonds to both the thione and thia­zole S atoms. The third I atom serves to link neighboring chains through a weak C—I⋯S—C inter­action to a thia­zole S atom; however, the geometry of this inter­action [C—I⋯S = 149.3 (1) and 142.48 (13)°] is indicative of a dispersive Type I inter­action and not a true halogen or chalcogen bond. Finally, (**MBZTH**)·(**TIE**) crystallized in the triclinic space group *P* 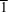 with one unique mol­ecule of **MBZTH** and two unique half mol­ecules of **TIE** in the asymmetric unit. Thio­amide hydro­gen-bonding dimers (Table 20 ▸) are linked into chains by C—I⋯S halogen bonding to the thione S atom. These chains are linked in the *ab* plane by additional C—I⋯S halogen bonding to the thione S atom. The second unique **TIE** mol­ecule serves to consolidate the packing in the *c* direction *via* C—I⋯I halogen bonding, forming a three-dimensional framework.

## Conclusion

A rich structural chemistry of cocrystals was observed between organoiodine mol­ecules and heterocyclic thio­nes in the present study of 18 crystal structures. The structures are primarily directed by the co-operative effects of hydrogen- and halogen-bonding inter­actions. Certain features of the long-range structures were controlled through the selection of the heterocyclic thione, where the formation of primarily hydrogen-bonded ribbons in benzimidazoles could be truncated to hydrogen-bonded dimers in benzoxazoles and benzo­thia­zoles. The hydrogen-bonded units were then aggregated into longer-range one- or two-dimensional motifs through C—I⋯S halogen bonding. Additional C—I⋯I halogen bonding, either through the stoichiometric excess of organoiodine or through the use of more iodine-rich organo­iodine substrates (tetra­iodo­ethyl­ene, for example) extended some structures into three-dimensional frameworks. The *R*_XB_ value for the majority of the halogen-bonding inter­actions lies within a typical range from 0.85 to 1.0. The inter­actions to a thione S atom generally occurred at shorter distances than the thiane S atom, as expected due to the hybridization state. The linearity parameter, ψ, ranges from 0.02 to 0.83. This wide range is supported by the distribution of electron density on S or I acceptor atoms. Occasional C=S⋯I chalcogen bonding was observed. Halogen-bond preference toward the thione S atom over the heterocyclic O or S atom was observed in both the benzoxazoles and benzo­thia­zoles. However, there were at least some occasional occurrences of C—I⋯S to the thia­zole S atom.

## Supplementary Material

Crystal structure: contains datablock(s) 2IT_13F4DIB, IT_135F3I3B, 4MBZIM_313F4DIB, MBZIM_14F4DIB, MBZIM_TIE, MMBZIM_12F4DIB, 2MMBZIM_14F4DIB_2H2O, MMBZIM_135F3I3B, MBZOX_12F4DIB, MBZOX_13F4DIB, 2MBZOX_14F4DIB, MBZOX_135F3I3B, 3MBZTH_412F4DIB, MBZTH_13F4DIB, MBZTH_213F4DIB, 2MBZTH_14F4DIB, MBZTH_135F3I3B, MBZTH_TIE, global. DOI: 10.1107/S2053229622009548/qw3002sup1.cif

Structure factors: contains datablock(s) 2IT_13F4DIB. DOI: 10.1107/S2053229622009548/qw30022IT_13F4DIBsup2.hkl

Structure factors: contains datablock(s) IT_135F3I3B. DOI: 10.1107/S2053229622009548/qw3002IT_135F3I3Bsup3.hkl

Structure factors: contains datablock(s) 4MBZIM_313F4DIB. DOI: 10.1107/S2053229622009548/qw30024MBZIM_313F4DIBsup4.hkl

Structure factors: contains datablock(s) MBZIM_14F4DIB. DOI: 10.1107/S2053229622009548/qw3002MBZIM_14F4DIBsup5.hkl

Structure factors: contains datablock(s) MBZIM_TIE. DOI: 10.1107/S2053229622009548/qw3002MBZIM_TIEsup6.hkl

Structure factors: contains datablock(s) MMBZIM_12F4DIB. DOI: 10.1107/S2053229622009548/qw3002MMBZIM_12F4DIBsup7.hkl

Structure factors: contains datablock(s) 2MMBZIM_14F4DIB_2H2O. DOI: 10.1107/S2053229622009548/qw30022MMBZIM_14F4DIB_2H2Osup8.hkl

Structure factors: contains datablock(s) MMBZIM_135F3I3B. DOI: 10.1107/S2053229622009548/qw3002MMBZIM_135F3I3Bsup9.hkl

Structure factors: contains datablock(s) MBZOX_12F4DIB. DOI: 10.1107/S2053229622009548/qw3002MBZOX_12F4DIBsup10.hkl

Structure factors: contains datablock(s) MBZOX_13F4DIB. DOI: 10.1107/S2053229622009548/qw3002MBZOX_13F4DIBsup11.hkl

Structure factors: contains datablock(s) 2MBZOX_14F4DIB. DOI: 10.1107/S2053229622009548/qw30022MBZOX_14F4DIBsup12.hkl

Structure factors: contains datablock(s) MBZOX_135F3I3B. DOI: 10.1107/S2053229622009548/qw3002MBZOX_135F3I3Bsup13.hkl

Structure factors: contains datablock(s) 3MBZTH_412F4DIB. DOI: 10.1107/S2053229622009548/qw30023MBZTH_412F4DIBsup14.hkl

Structure factors: contains datablock(s) MBZTH_13F4DIB. DOI: 10.1107/S2053229622009548/qw3002MBZTH_13F4DIBsup15.hkl

Structure factors: contains datablock(s) MBZTH_213F4DIB. DOI: 10.1107/S2053229622009548/qw3002MBZTH_213F4DIBsup16.hkl

Structure factors: contains datablock(s) 2MBZTH_14F4DIB. DOI: 10.1107/S2053229622009548/qw30022MBZTH_14F4DIBsup17.hkl

Structure factors: contains datablock(s) MBZTH_135F3I3B. DOI: 10.1107/S2053229622009548/qw3002MBZTH_135F3I3Bsup18.hkl

Structure factors: contains datablock(s) MBZTH_TIE. DOI: 10.1107/S2053229622009548/qw3002MBZTH_TIEsup19.hkl

Supporting information file. DOI: 10.1107/S2053229622009548/qw30022IT_13F4DIBsup20.cml

Supporting information file. DOI: 10.1107/S2053229622009548/qw3002IT_135F3I3Bsup21.cml

Supporting information file. DOI: 10.1107/S2053229622009548/qw30024MBZIM_313F4DIBsup22.cml

Supporting information file. DOI: 10.1107/S2053229622009548/qw3002MBZIM_14F4DIBsup23.cml

Supporting information file. DOI: 10.1107/S2053229622009548/qw3002MBZIM_TIEsup24.cml

Supporting information file. DOI: 10.1107/S2053229622009548/qw3002MMBZIM_12F4DIBsup25.cml

Supporting information file. DOI: 10.1107/S2053229622009548/qw30022MMBZIM_14F4DIB_2H2Osup26.cml

Supporting information file. DOI: 10.1107/S2053229622009548/qw3002MMBZIM_135F3I3Bsup27.cml

Supporting information file. DOI: 10.1107/S2053229622009548/qw3002MBZOX_12F4DIBsup28.cml

Supporting information file. DOI: 10.1107/S2053229622009548/qw3002MBZOX_13F4DIBsup29.cml

Supporting information file. DOI: 10.1107/S2053229622009548/qw30022MBZOX_14F4DIBsup30.cml

Supporting information file. DOI: 10.1107/S2053229622009548/qw3002MBZOX_135F3I3Bsup31.cml

Supporting information file. DOI: 10.1107/S2053229622009548/qw30023MBZTH_412F4DIBsup32.cml

Supporting information file. DOI: 10.1107/S2053229622009548/qw3002MBZTH_13F4DIBsup33.cml

Supporting information file. DOI: 10.1107/S2053229622009548/qw3002MBZTH_213F4DIBsup34.cml

Supporting information file. DOI: 10.1107/S2053229622009548/qw30022MBZTH_14F4DIBsup35.cml

Supporting information file. DOI: 10.1107/S2053229622009548/qw3002MBZTH_135F3I3Bsup36.cml

Supporting information file. DOI: 10.1107/S2053229622009548/qw3002MBZTH_TIEsup37.cml

CCDC references: 2194060, 2194061, 2194062, 2194063, 2194064, 2194065, 2194066, 2194067, 2194068, 2194069, 2194070, 2194071, 2194072, 2194073, 2194074, 2194075, 2194076, 2194077

## Figures and Tables

**Table:** Experiments were carried out at 100 K with Mo *K*α radiation using a Bruker D8 Venture Photon 2 diffractometer. Absorption was corrected for by multi-scan methods (*SADABS*; Bruker, 2017 ▸). H atoms were treated by a mixture of independent and constrained refinement, except for 3(**MBZTH**)·4(**1,2-F_4_DIB**), for which H-atom parameters were constrained.

	2(**IT**)·(**1,3-F_4_DIB**)	(**IT**)·(**1,3,5-F_3_I_3_B**)	4(**MBZIM**)·3(**1,3-F_4_DIB**)	(**MBZIM**)·(**1,4-F_4_DIB**)
Crystal data				
Chemical formula	C_6_F_4_I_2_·2C_3_H_6_N_2_S	C_6_F_3_I_3_·C_3_H_6_N_2_S	3C_6_F_4_I_2_·4C_7_H_6_N_2_S	C_6_F_4_I_2_·C_7_H_6_N_2_S
*M*_r_	606.18	611.92	1806.37	552.06
Crystal system, space group	Orthorhombic, *P* *b* *c* *n*	Orthorhombic, *P* *b* *c* *a*	Triclinic, *P* 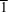	Monoclinic, *P*2_1_/*c*
*a*, *b*, *c* (Å)	15.6704 (7), 8.9924 (4), 26.0573 (10)	18.0407 (14), 7.2816 (6), 22.1250 (19)	8.4573 (14), 17.725 (3), 18.759 (4)	5.5641 (2), 33.1320 (11), 8.4710 (3)
α, β, γ (°)	90, 90, 90	90, 90, 90	106.997 (7), 93.229 (7), 92.034 (7)	90, 92.754 (1), 90
*V* (Å^3^)	3671.9 (3)	2906.5 (4)	2680.9 (9)	1559.82 (9)
*Z*	8	8	2	4
μ (mm^−1^)	3.69	6.61	3.72	4.20
Crystal size (mm)	0.18 × 0.17 × 0.13	0.22 × 0.08 × 0.04	0.34 × 0.04 × 0.04	0.22 × 0.18 × 0.06

Data collection				
*T*_min_, *T*_max_	0.639, 0.746	0.563, 0.746	0.668, 0.746	0.501, 0.746
No. of measured, independent and observed [*I* > 2σ(*I*)] reflections	112821, 5376, 5198	49179, 3615, 3220	118524, 12297, 10558	45583, 4579, 4211
*R*_int_	0.035	0.043	0.056	0.050
(sin θ/λ)_max_ (Å^−1^)	0.705	0.667	0.651	0.709

Refinement				
*R*[*F* ^2^ > 2σ(*F* ^2^)], *wR*(*F* ^2^), *S*	0.015, 0.032, 1.25	0.019, 0.040, 1.11	0.021, 0.041, 1.06	0.020, 0.044, 1.12
No. of reflections	5376	3615	12297	4579
No. of parameters	234	172	717	207
No. of restraints	0	2	8	0
Δρ_max_, Δρ_min_ (e Å^−3^)	0.42, −0.37	0.57, −0.77	0.52, −0.75	0.49, −0.68

**Table:** 

	(**MBZIM**)·(**TIE**)	(**MMBZIM**)·(**1,2-F_4_DIB**)	2(**MMBZIM**)·(**1,4-F_4_DIB**)·2(**H_2_O**)	(**MMBZIM**)·(**1,3,5-F_3_I_3_B**)
Crystal data				
Chemical formula	C_2_I_4_·C_7_H_6_N_2_S	C_6_F_4_I_2_·C_8_H_8_N_2_S	C_6_F_4_I_2_·2C_8_H_8_N_2_S·2(H_2_O)	C_6_F_3_I_3_·C_8_H_8_N_2_S
*M*_r_	681.82	566.08	766.34	673.98
Crystal system, space group	Orthorhombic, *P* *n* *m* *a*	Triclinic, *P* 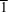	Triclinic, *P* 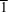	Monoclinic, *P*2_1_/*c*
*a*, *b*, *c* (Å)	11.7547 (10), 8.3525 (7), 15.1077 (13)	4.5504 (5), 13.2872 (14), 13.8064 (14)	4.9088 (3), 11.4670 (8), 11.9686 (8)	15.191 (2), 5.0074 (7), 22.715 (3)
α, β, γ (°)	90, 90, 90	94.766 (4), 98.124 (4), 99.588 (4)	106.644 (2), 98.058 (2), 92.811 (2)	90, 97.460 (6), 90
*V* (Å^3^)	1483.3 (2)	809.97 (15)	636.27 (7)	1713.3 (4)
*Z*	4	2	1	4
μ (mm^−1^)	8.52	4.05	2.69	5.62
Crystal size (mm)	0.30 × 0.14 × 0.11	0.19 × 0.07 × 0.04	0.31 × 0.11 × 0.08	0.26 × 0.04 × 0.04

Data collection				
*T*_min_, *T*_max_	0.256, 0.746	0.636, 0.746	0.536, 0.746	0.582, 0.746
No. of measured, independent and observed [*I* > 2σ(*I*)] reflections	32859, 1993, 1885	21426, 3704, 3174	31584, 3558, 3500	23258, 3971, 3039
*R*_int_	0.055	0.042	0.036	0.069
(sin θ/λ)_max_ (Å^−1^)	0.671	0.650	0.696	0.652

Refinement				
*R*[*F* ^2^ > 2σ(*F* ^2^)], *wR*(*F* ^2^), *S*	0.026, 0.062, 1.26	0.026, 0.055, 1.24	0.014, 0.034, 1.18	0.047, 0.105, 1.22
No. of reflections	1993	3704	3558	3971
No. of parameters	89	217	184	217
No. of restraints	0	1	7	1
Δρ_max_, Δρ_min_ (e Å^−3^)	1.25, −1.48	1.33, −1.06	0.44, −0.42	2.37, −1.89

**Table:** 

	(**MBZOX**)·(**1,2-F_4_DIB**)	(**MBZOX**)·(**1,3-F_4_DIB**)	2(**MBZOX**)·(**1,4-F_4_DIB**)	(**MBZOX**)·(**1,3,5-F_3_I_3_B**)
Crystal data				
Chemical formula	C_6_F_4_I_2_·C_7_H_5_NOS	C_6_F_4_I_2_·C_7_H_5_NOS	C_6_F_4_I_2_·2C_7_H_5_NOS	C_6_F_3_I_3_·C_7_H_5_NOS
*M*_r_	553.04	553.04	704.22	660.94
Crystal system, space group	Monoclinic, *P*2_1_/*n*	Monoclinic, *P*2_1_/*c*	Monoclinic, *C*2/*c*	Monoclinic, *P*2_1_/*c*
*a*, *b*, *c* (Å)	13.7789 (12), 4.4129 (4), 25.252 (2)	15.1655 (8), 4.3803 (2), 23.0358 (12)	31.025 (4), 4.3159 (5), 19.061 (2)	14.9295 (7), 4.6119 (2), 23.5065 (12)
α, β, γ (°)	90, 96.337 (3), 90	90, 99.923 (2), 90	90, 114.434 (4), 90	90, 92.548 (2), 90
*V* (Å^3^)	1526.0 (2)	1507.36 (13)	2323.6 (5)	1616.90 (13)
*Z*	4	4	4	4
μ (mm^−1^)	4.30	4.35	2.94	5.96
Crystal size (mm)	0.46 × 0.06 × 0.02	0.23 × 0.12 × 0.09	0.29 × 0.12 × 0.03	0.22 × 0.06 × 0.05

Data collection				
*T*_min_, *T*_max_	0.578, 0.745	0.541, 0.746	0.637, 0.746	0.551, 0.745
No. of measured, independent and observed [*I* > 2σ(*I*)] reflections	12498, 3210, 2510	39610, 4625, 4119	25197, 2950, 2571	19413, 3348, 2845
*R*_int_	0.066	0.042	0.047	0.050
(sin θ/λ)_max_ (Å^−1^)	0.634	0.716	0.675	0.630

Refinement				
*R*[*F* ^2^ > 2σ(*F* ^2^)], *wR*(*F* ^2^), *S*	0.047, 0.087, 1.11	0.022, 0.048, 1.16	0.028, 0.060, 1.32	0.029, 0.061, 1.22
No. of reflections	3210	4625	2950	3348
No. of parameters	203	203	149	203
No. of restraints	0	0	0	0
Δρ_max_, Δρ_min_ (e Å^−3^)	1.58, −1.52	0.96, −1.35	1.54, −1.15	0.80, −0.77

**Table:** 

	3(**MBZTH**)·4(**1,2-F_4_DIB**)	(**MBZTH**)·(**1,3-F_4_DIB**)	(**MBZTH**)·2(**1,3-F_4_DIB**)	2(**MBZTH**)·(**1,4-F_4_DIB**)
Crystal data				
Chemical formula	4C_6_F_4_I_2_·3C_7_H_5_NS_2_	C_6_F_4_I_2_·C_7_H_5_NS_2_	4C_6_F_4_I_2_·2C_7_H_5_NS_2_	C_6_F_4_I_2_·2C_7_H_5_NS_2_
*M*_r_	2109.16	569.10	1941.92	736.34
Crystal system, space group	Triclinic, *P* 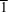	Triclinic, *P* 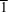	Monoclinic, *P*2_1_	Monoclinic, *P*2_1_/*n*
*a*, *b*, *c* (Å)	7.9410 (8), 14.8483 (15), 24.641 (3)	7.2175 (4), 8.2675 (5), 14.4498 (9)	4.5581 (3), 34.358 (2), 15.6075 (10)	5.5057 (2), 15.6087 (7), 13.5194 (6)
α, β, γ (°)	79.264 (4), 87.104 (4), 82.784 (4)	97.936 (2), 91.297 (2), 109.178 (2)	90, 94.707 (2), 90	90, 94.259 (2), 90
*V* (Å^3^)	2830.9 (5)	804.44 (8)	2436.0 (3)	1158.61 (8)
*Z*	2	2	2	2
μ (mm^−1^)	4.69	4.20	5.36	3.12
Crystal size (mm)	0.30 × 0.13 × 0.04	0.33 × 0.27 × 0.06	0.18 × 0.12 × 0.04	0.17 × 0.09 × 0.04

Data collection				
*T*_min_, *T*_max_	0.570, 0.746	0.496, 0.746	0.568, 0.746	0.559, 0.746
No. of measured, independent and observed [*I* > 2σ(*I*)] reflections	78566, 12466, 11325	27899, 4701, 4391	56285, 12660, 11766	22270, 3402, 2811
*R*_int_	0.067	0.036	0.050	0.049
(sin θ/λ)_max_ (Å^−1^)	0.642	0.706	0.678	0.706

Refinement				
*R*[*F* ^2^ > 2σ(*F* ^2^)], *wR*(*F* ^2^), *S*	0.067, 0.220, 1.06	0.018, 0.044, 1.09	0.026, 0.046, 1.09	0.027, 0.057, 1.15
No. of reflections	12466	4701	12660	3402
No. of parameters	704	203	622	149
No. of restraints	66	0	2	0
Δρ_max_, Δρ_min_ (e Å^−3^)	2.61, −1.48	1.08, −1.11	1.01, −0.71	0.90, −0.79
Absolute structure	–	–	Refined as an inversion twin	–
Absolute structure parameter	–	–	0.454 (15)	–

**Table:** 

	(**MBZTH**)·(**1,3,5-F_3_I_3_B**)	(**MBZTH**)·(**TIE**)
Crystal data		
Chemical formula	C_6_F_3_I_3_·C_7_H_5_NS_2_	C_2_I_4_·C_7_H_5_NS_2_
*M*_r_	677.00	698.86
Crystal system, space group	Monoclinic, *P*2_1_/*c*	Triclinic, *P* 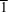
*a*, *b*, *c* (Å)	15.2665 (6), 4.7380 (2), 23.2215 (10)	7.4085 (6), 10.8180 (9), 11.1989 (10)
α, β, γ (°)	90, 93.139 (2), 90	66.616 (3), 70.765 (3), 70.792 (3)
*V* (Å^3^)	1677.15 (12)	757.20 (11)
*Z*	4	2
μ (mm^−1^)	5.86	8.48
Crystal size (mm)	0.16 × 0.08 × 0.05	0.08 × 0.07 × 0.07

Data collection		
*T*_min_, *T*_max_	0.610, 0.746	0.589, 0.746
No. of measured, independent and observed [*I* > 2σ(*I*)] reflections	35222, 4212, 3611	22463, 3484, 3037
*R*_int_	0.057	0.052
(sin θ/λ)_max_ (Å^−1^)	0.669	0.651

Refinement		
*R*[*F* ^2^ > 2σ(*F* ^2^)], *wR*(*F* ^2^), *S*	0.024, 0.052, 1.18	0.029, 0.072, 1.13
No. of reflections	4212	3484
No. of parameters	203	159
No. of restraints	1	7
Δρ_max_, Δρ_min_ (e Å^−3^)	0.67, −0.73	1.43, −1.76

com­puter programs: *APEX3* (Bruker, 2017 ▸), *SAINT* (Bruker, 2017 ▸), *SHELXT2018* (Sheldrick, 2015*a* ▸), *SHELXL2018* (Sheldrick, 2015*b* ▸), *Mercury* (Macrae *et al.*, 2020 ▸), and *OLEX2* (Dolomanov *et al.*, 2009 ▸).

**Table 2:** Hydrogen-bond geometry (Å, °) for 2(**IT**)·(**1,3-F_4_DIB**)

*D*—H⋯*A*	*D*—H	H⋯*A*	*D*⋯*A*	*D*—H⋯*A*
N1—HN1⋯S1^i^	0.81 (2)	2.77 (2)	3.5551 (14)	163 (2)
N2—HN2⋯S2^ii^	0.83 (2)	2.53 (2)	3.3507 (14)	172 (2)
C2—H2*B*⋯F2	0.99	2.55	3.3392 (19)	136
C3—H3*B*⋯S2	0.99	2.94	3.7351 (19)	138
N3—HN3⋯S2^iii^	0.79 (2)	2.54 (2)	3.3171 (15)	167 (2)
N4—HN4⋯I2^iv^	0.83 (2)	3.31 (2)	3.7383 (14)	114.9 (18)
N4—HN4⋯S1^ii^	0.83 (2)	2.63 (2)	3.4562 (14)	179 (2)
C5—H5*A*⋯I1^v^	0.99	3.20	3.9922 (16)	138
C5—H5*B*⋯F4	0.99	2.45	3.2774 (19)	140
C6—H6*B*⋯I1^vi^	0.99	3.18	3.9223 (16)	133

Symmetry codes: (i) 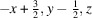 ; (ii) 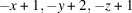 ; (iii) 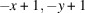 , 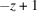 ; (iv) 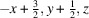 ; (v) 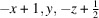 ; (vi) 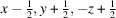 .

**Table 3:** Halogen- and chalcogen-bond geometries (Å, °)

Compound		*d*(*D*⋯*A*)	*R*_XB_^i^	θ(C—*D*⋯*A*)	θ(*D*⋯*A*—C)	θ_1_ – θ_2_^ii^	ψ^iii^
2(**IT**)·(**1,3-F_4_DIB**)	I1⋯S1	3.2265 (6)	0.85	174.51 (4)	113.51 (5)	61.00	0.79
	I2⋯S1	3.2860 (5)	0.87	176.10 (4)	99.67 (5)	76.43	0.03
(**IT**)·(**1,3,5-F_3_I_3_B**)	I1⋯I3	3.8376 (6)	0.97	162.95 (8)	106.24 (7)	56.71	0.64
	I2⋯S1	3.1505 (8)	0.83	171.86 (7)	101.89 (10)	69.97	0.48
	I3⋯S1	3.1754 (8)	0.84	177.68 (8)	90.59 (9)	87.09	0.18
4(**MBZIM**)·3(**1,3-F_4_DIB**)	I1⋯S3	3.3361 (10)	0.88	172.18 (8)	136.86 (8)	35.32	0.83
	I6⋯S2	3.2150 (9)	0.85	166.06 (8)	134.24 (8)	31.82	0.74
(**MBZIM**)·(**1,4-F_4_DIB**)	I1⋯S1	3.2573 (9)	0.86	168.29 (6)	131.28 (8)	37.01	0.57
(**MBZIM**)·(**TIE**)	I1⋯S1	3.5368 (14)	0.94	173.83 (17)	71.37 (16)	102.46	0.66
	I3⋯S1	3.2702 (14)	0.87	177.05 (17)	118.15 (17)	58.90	0.64
(**MMBZIM**)·(**1,2-F_4_DIB**)	I1⋯S1	3.6404 (10)	0.96	154.63 (12)	95.76 (13)	58.87	0.47
	I2⋯S1	3.2307 (11)	0.85	169.63 (11)	106.66 (15)	62.97	0.04
2(**MMBZIM**)·(**1,4-F_4_DIB**)·2(**H_2_O**)	I1⋯S1	3.2516 (4)	0.86	169.16 (4)	96.12 (4)	73.04	0.37
(**MMBZIM**)·(**1,3,5-F_3_I_3_B**)	I2⋯S1	3.474 (2)	0.92	164.1 (3)	92.1 (3)	72.0	0.71
	I3⋯S1	3.463 (2)	0.92	176.7 (3)	96.5 (3)	80.2	0.50
(**MBZOX**)·(**1,2-F_4_DIB**)	I2⋯S1	3.2853 (19)	0.87	166.65 (19)	105.4 (3)	61.3	0.19
(**MBZOX**)·(**1,3-F_4_DIB**)	I1⋯S1	3.4132 (7)	0.90	174.53 (6)	105.63 (8)	68.90	0.19
	I2⋯S1	3.6787 (6)	0.97	159.40 (6)	92.48 (7)	66.92	0.66
	S1⋯I1	3.7536 (7)	0.99	160.34 (7)	105.80 (6)	54.54	0.53
2(**MBZOX**)·(**1,4-F_4_DIB**)	I1⋯S1	3.2287 (11)	0.85	174.60 (9)	109.39 (13)	65.21	0.74
(**MBZOX**)·(**1,3,5-F_3_I_3_B**)	I1⋯S1	3.4114 (14)	0.90	171.55 (13)	100.14 (19)	71.41	0.14
	I2⋯I3	3.9110 (7)	0.99	147.22 (13)	79.55 (13)	67.67	0.62
	I3⋯S1	3.6774 (13)	0.97	157.67 (15)	95.79 (16)	61.88	0.67
	S1⋯I1	3.7385 (14)	0.99	163.12 (17)	98.99 (14)	64.13	0.44
3(**MBZTH**)·4(**1,2-F_4_DIB**)	I1⋯S5	3.380 (4)	0.89	177.9 (4)	113.3 (5)	64.6	0.81
	I2⋯S5	3.353 (4)	0.89	163.7 (4)	128.9 (5)	34.8	0.61
	I3⋯S3	3.371 (5)	0.89	169.0 (4)	96.4 (7)	72.6	0.02
	I4⋯S3	3.754 (4)	0.99	173.7 (4)	100.5 (6)	73.2	0.76
	I5⋯S1	3.380 (4)	0.89	177.9 (4)	113.3 (5)	64.6	0.81
	I6⋯I7	3.8766 (14)	0.95	170.5 (4)	118.1 (4)	52.4	0.73
	I6⋯S1	3.391 (5)	0.90	168.1 (4)	111.3 (6)	56.8	0.76
(**MBZTH**)·(**1,3-F_4_DIB**)	I1⋯S1	3.3724 (5)	0.89	168.06 (6)	120.18 (6)	47.88	0.64
	I2⋯S1	3.4140 (5)	0.90	157.68 (4)	106.00 (5)	51.68	0.66
(**MBZTH**)·2(**1,3-F_4_DIB**)	I1⋯S3	3.3426 (17)	0.88	168.34 (18)	103.3 (2)	65.0	0.25
	I2⋯S2	3.7429 (17)	0.99	152.94 (18)	121.7 (4)^iii^	31.2	0.23
	I3⋯S1	3.3548 (18)	0.89	166.60 (17)	100.3 (2)	66.3	0.64
	I4⋯S4	3.6744 (17)	0.97	148.74 (18)	118.4 (4)^iii^	30.4	0.65
	I5⋯I4	3.7971 (10)	0.96	163.30 (19)	82.92 (18)	80.38	0.52
	I8⋯I2	3.7950 (9)	0.96	170.03 (18)	84.69 (18)	85.34	0.71
2(**MBZTH**)·(**1,4-F_4_DIB**)	I1⋯S1	3.3013 (7)	0.87	178.16 (7)	103.84 (10)	74.32	0.60
(**MBZTH**)·(**1,3,5-F_3_I_3_B**)	I1⋯S1	3.4551 (10)	0.91	169.01 (9)	97.89 (13)	71.12	0.71
	S2⋯I3	3.7777 (10)	1.00	158.73 (12)	117.64 (10)	41.09	0.52
(**MBZTH**)·(**TIE**)	I1⋯I3	3.9459 (7)	1.00	171.07 (14)	70.7 (3)	100.4	0.55
	I3⋯S1	3.2826 (13)	0.87	162.1 (3)	122.51 (19)	39.6	0.41
	I4⋯S1	3.6514 (19)	0.97	161.9 (3)	77.0 (2)	84.9	0.46

Notes: (i) *R*_XB_ = *d*(*X*⋯*Y*)/Σ*d*(vdW), the ratio of the distance between the donor atom (*i.e.* I) and the acceptor atom (*i.e.* S) to the sum of their van der Waals (vdW) radii (S = 1.80 Å and I = 1.98 Å) (Auffinger *et al.*, 2004 ▸). (ii) θ_1_ – θ_2_ = |{[θ(C—*D*⋯*A*)] – [θ(*D*⋯*A*—C)]}|. (iii) Linearity parameter (Setter *et al.*, 2020 ▸).

**Table 4:** Hydrogen-bond geometry (Å, °) for (**IT**)·(**1,3,5-F_3_I_3_B**)

*D*—H⋯*A*	*D*—H	H⋯*A*	*D*⋯*A*	*D*—H⋯*A*
N2—HN2⋯I2^i^	0.83 (2)	3.10 (3)	3.742 (3)	137 (3)
C2—H2*B*⋯I1^ii^	0.99	3.31	3.927 (3)	122
C2—H2*B*⋯F3^iii^	0.99	2.47	3.147 (3)	125

Symmetry codes: (i) 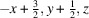 ; (ii) 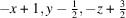 ; (iii) 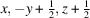 .

**Table 5:** Hydrogen-bond geometry (Å, °) for 4(**MBZIM**)·3(**1,3-F_4_DIB**)

*D*—H⋯*A*	*D*—H	H⋯*A*	*D*⋯*A*	*D*—H⋯*A*
N1—HN1⋯S2^i^	0.85 (2)	2.52 (2)	3.357 (2)	172 (3)
N2—HN2⋯S2	0.85 (2)	2.46 (2)	3.297 (2)	166 (2)
N3—HN3⋯S1	0.85 (2)	2.51 (2)	3.348 (2)	173 (3)
N4—HN4⋯S1^ii^	0.85 (2)	2.50 (2)	3.326 (2)	166 (2)
N5—HN5⋯S4^i^	0.85 (2)	2.49 (2)	3.326 (2)	169 (3)
N6—HN6⋯S4	0.85 (2)	2.43 (2)	3.270 (2)	169 (3)
C17—H17⋯F36^iii^	0.95	2.61	3.385 (3)	139
C20—H20⋯F36^iv^	0.95	2.51	3.235 (3)	133
N7—HN7⋯S3	0.84 (2)	2.47 (2)	3.300 (2)	170 (3)
N8—HN8⋯S3^ii^	0.85 (2)	2.48 (2)	3.302 (2)	163 (3)

Symmetry codes: (i) 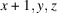 ; (ii) 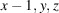 ; (iii) 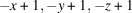 ; (iv) 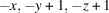 .

**Table 6:** Hydrogen-bond geometry (Å, °) for (**MBZIM**)·(**1,4-F_4_DIB**)

*D*—H⋯*A*	*D*—H	H⋯*A*	*D*⋯*A*	*D*—H⋯*A*
N1—HN1⋯S1^i^	0.84 (3)	2.47 (3)	3.3089 (18)	172 (2)
N2—HN2⋯S1^ii^	0.86 (3)	2.50 (3)	3.3527 (17)	172 (2)

Symmetry codes: (i) 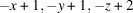 ; (ii) 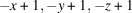 .

**Table 7:** Hydrogen-bond geometry (Å, °) for (**MBZIM**)·(**TIE**)

*D*—H⋯*A*	*D*—H	H⋯*A*	*D*⋯*A*	*D*—H⋯*A*
N1—HN1⋯S1^i^	0.87 (5)	2.47 (5)	3.335 (3)	178 (5)
C3—H3⋯I1^ii^	0.95	3.28	3.881 (4)	123

Symmetry codes: (i) 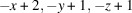 ; (ii) 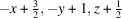 .

**Table 8:** Hydrogen-bond geometry (Å, °) for (**MMBZIM**)·(**1,2-F_4_DIB**)

*D*—H⋯*A*	*D*—H	H⋯*A*	*D*⋯*A*	*D*—H⋯*A*
N1—HN1⋯S1^i^	0.88 (5)	2.57 (5)	3.444 (3)	173 (4)
N2—HN2⋯I1	0.85 (2)	3.07 (3)	3.780 (3)	142 (3)
N2—HN2⋯F4	0.85 (2)	2.56 (3)	3.122 (4)	124 (3)
C3—H3⋯I2^ii^	0.95	3.06	3.966 (4)	160
C6—H6⋯F4	0.95	2.63	3.262 (4)	125

Symmetry codes: (i) 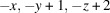 ; (ii) 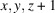 .

**Table 9:** Hydrogen-bond geometry (Å, °) for 2(**MMBZIM**)·(**1,4-F_4_DIB**)·2(**H_2_O**)

*D*—H⋯*A*	*D*—H	H⋯*A*	*D*⋯*A*	*D*—H⋯*A*
N1—HN1⋯O1	0.83 (2)	2.06 (2)	2.8763 (17)	166 (2)
N2—HN2⋯S1^i^	0.88 (2)	2.57 (2)	3.4211 (13)	164 (2)
C4—H4⋯I1^ii^	0.95	3.03	3.9505 (14)	164
O1—H1*A*O⋯O1^iii^	0.88 (2)	1.85 (2)	2.708 (3)	163 (4)
O1—H1*B*O⋯O1^iv^	0.88 (2)	1.89 (2)	2.759 (3)	167 (4)
O1—H2O1⋯I1	0.87 (2)	3.16 (3)	3.7419 (12)	126 (2)
O1—H2O1⋯S1^iii^	0.87 (2)	2.65 (2)	3.4251 (13)	149 (3)

Symmetry codes: (i) 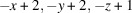 ; (ii) 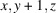 ; (iii) 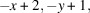 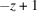 ; (iv) 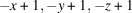 .

**Table 10:** Hydrogen-bond geometry (Å, °) for (**MMBZIM**)·(**1,3,5-F_3_I_3_B**)

*D*—H⋯*A*	*D*—H	H⋯*A*	*D*⋯*A*	*D*—H⋯*A*
N1—HN1⋯S1^i^	0.86 (2)	2.57 (3)	3.426 (7)	173 (10)
N2—HN2⋯I2^ii^	0.85 (8)	3.02 (8)	3.657 (7)	133 (7)
C3—H3⋯I3^iii^	0.95	3.12	4.035 (9)	163
C6—H6⋯I1^iv^	0.95	3.14	3.927 (8)	142

Symmetry codes: (i) 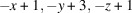 ; (ii) 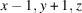 ; (iii) 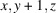 ; (iv) 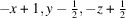 .

**Table 11:** Hydrogen-bond geometry (Å, °) for (**MBZOX**)·(**1,2-F_4_DIB**)

*D*—H⋯*A*	*D*—H	H⋯*A*	*D*⋯*A*	*D*—H⋯*A*
N1—HN1⋯S1^i^	0.85 (8)	2.50 (8)	3.335 (6)	167 (8)
C3—H3⋯I2^ii^	0.95	3.19	4.108 (7)	162

Symmetry codes: (i) 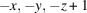 ; (ii) 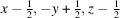 .

**Table 12:** Hydrogen-bond geometry (Å, °) for (**MBZOX**)·(**1,3-F_4_DIB**)

*D*—H⋯*A*	*D*—H	H⋯*A*	*D*⋯*A*	*D*—H⋯*A*
N1—HN1⋯S1^i^	0.88 (3)	2.52 (3)	3.3906 (19)	172 (3)
C3—H3⋯I1^ii^	0.95	3.10	4.030 (2)	166

Symmetry codes: (i) 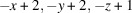 ; (ii) 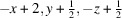 .

**Table 13:** Hydrogen-bond geometry (Å, °) for 2(**MBZOX**)·(**1,4-F_4_DIB**)

*D*—H⋯*A*	*D*—H	H⋯*A*	*D*⋯*A*	*D*—H⋯*A*
N1—HN1⋯S1^i^	0.87 (4)	2.45 (4)	3.316 (3)	178 (4)
C3—H3⋯I1^ii^	0.95	3.16	4.066 (3)	159

Symmetry codes: (i) 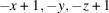 ; (ii) 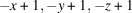 .

**Table 14:** Hydrogen-bond geometry (Å, °) for (**MBZOX**)·(**1,3,5-F_3_I_3_B**)

*D*—H⋯*A*	*D*—H	H⋯*A*	*D*⋯*A*	*D*—H⋯*A*
N1—HN1⋯S1^i^	0.85 (7)	2.53 (7)	3.377 (4)	176 (6)
C3—H3⋯I1^ii^	0.95	3.04	3.969 (5)	167
C6—H6⋯I2^iii^	0.95	3.23	4.009 (5)	140

Symmetry codes: (i) 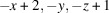 ; (ii) 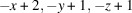 ; (iii) 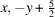 , 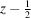 .

**Table 15:** Hydrogen-bond geometry (Å, °) for 3(**MBZTH**)·4(**1,2-F_4_DIB**)

*D*—H⋯*A*	*D*—H	H⋯*A*	*D*⋯*A*	*D*—H⋯*A*
N2—HN2⋯S1	0.88	2.45	3.326 (14)	174
N1—HN1⋯S3	0.88	2.40	3.266 (14)	169
C6—H6⋯F10^i^	0.95	2.60	3.29 (2)	130
N3—HN3⋯S5^ii^	0.88	2.42	3.290 (14)	170
C17—H17⋯F16	0.95	2.30	3.232 (18)	166
C20—H20⋯F2	0.95	2.53	3.128 (18)	121
C20—H20⋯F3	0.95	2.54	3.181 (17)	125

Symmetry codes: (i) 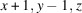 ; (ii) 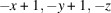 .

**Table 16:** Hydrogen-bond geometry (Å, °) for (**MBZTH**)·(**1,3-F_4_DIB**)

*D*—H⋯*A*	*D*—H	H⋯*A*	*D*⋯*A*	*D*—H⋯*A*
N1—HN1⋯S1^i^	0.87 (3)	2.45 (3)	3.3120 (15)	175 (2)

Symmetry code: (i) 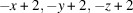 .

**Table 17:** Hydrogen-bond geometry (Å, °) for (**MBZTH**)·2(**1,3-F_4_DIB**)

*D*—H⋯*A*	*D*—H	H⋯*A*	*D*⋯*A*	*D*—H⋯*A*
N1—HN1⋯S3	0.89 (6)	2.51 (6)	3.376 (6)	165 (5)
C3—H3⋯I1	0.95	3.10	3.976 (7)	154
N2—HN2⋯S1	0.85 (3)	2.52 (3)	3.360 (6)	169 (7)
C10—H10⋯I3^i^	0.95	3.09	4.006 (6)	161

Symmetry code: (i) 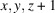 .

**Table 18:** Hydrogen-bond geometry (Å, °) for 2(**MBZTH**)·(**1,4-F_4_DIB**)

*D*—H⋯*A*	*D*—H	H⋯*A*	*D*⋯*A*	*D*—H⋯*A*
N1—HN1⋯S1^i^	0.78 (3)	2.60 (3)	3.369 (2)	170 (3)
C3—H3⋯F1^ii^	0.95	2.50	3.333 (3)	146
C6—H6⋯F2^iii^	0.95	2.44	3.357 (3)	162

Symmetry codes: (i) 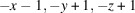 ; (ii) 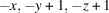 ; (iii) 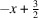 , 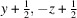 .

**Table 19:** Hydrogen-bond geometry (Å, °) for (**MBZTH**)·(**1,3,5-F_3_I_3_B**)

*D*—H⋯*A*	*D*—H	H⋯*A*	*D*⋯*A*	*D*—H⋯*A*
N1—HN1⋯S1^i^	0.86 (2)	2.54 (2)	3.389 (3)	172 (4)
C3—H3⋯I1^ii^	0.95	3.03	3.928 (4)	159

Symmetry codes: (i) 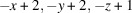 ; (ii) 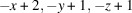 .

**Table 20:** Hydrogen-bond geometry (Å, °) for (**MBZTH**)·(**TIE**)

*D*—H⋯*A*	*D*—H	H⋯*A*	*D*⋯*A*	*D*—H⋯*A*
N1—HN1⋯S1^i^	0.85 (2)	2.43 (2)	3.275 (5)	170 (6)

Symmetry code: (i) 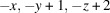 .
